# Peptides derived from cadherin juxtamembrane region inhibit platelet function

**DOI:** 10.1098/rsos.172347

**Published:** 2018-10-10

**Authors:** Kalyan Golla, Ilias Stavropoulos, Denis C. Shields, Niamh Moran

**Affiliations:** 1 Molecular and Cellular Therapeutics, Royal College of Surgeons in Ireland, Dublin, Ireland; 2 UCD Conway Institute of Biomolecular and Biomedical Research, University College Dublin, Dublin, Ireland; 3 School of Medicine and Medical Science, University College Dublin, Dublin, Ireland

**Keywords:** E-cadherin, N-cadherin, adherens junctions, juxtamembrane domain, peptides, P120-catenin

## Abstract

The juxtamembrane domains (JMD) of transmembrane proteins are rich in critical peptide sequences that participate in dynamic cell signalling events. Synthetic JMD peptides derived from cadherin cell adhesion proteins have previously been shown to modulate platelet function. In this study, we aimed to develop functional bioactive agents from bioinformatically identified critical peptide sequences. We synthesized overlapping 12–15 amino acid peptides from E- and N-cadherin JMD and assessed their effect on platelet aggregation and platelet ATP secretion. Peptides derived from close to the membrane proximal region inhibit platelet function. Sequential deletion of amino acids from the N- and C-termini of the inhibitory E-cadherin peptides identified the short K^756^EPLLP^763^ motif as a critical bioactive sequence. Alanine scanning studies further identified that the di-leucine (LL) motif and positively charged lysine (K) are crucial for peptide activity. Moreover, scrambled peptides failed to show any effect on platelet activity. We conclude that peptides derived from JMD of E-cadherin provide potential lead peptides for the development of anti-thrombotic agents and to enable further understanding of the role of cadherins in platelet function.

## 1. Introduction

Platelets are small anucleate blood cells that play an important role in haemostasis and thrombosis. Upon damage to a blood vessel, platelets become exposed to sub-endothelial matrix proteins or soluble platelet agonists. This process triggers platelet activation and contributes to formation of a platelet plug or thrombus at the damaged site. Unwanted activation of platelets within an intact blood vessel can result in the formation of a thrombus. Thrombosis underlies a number of cardiovascular disorders including angina, myocardial infarction, cardiac ischaemia and ischaemic stroke. As such, there is an ongoing search for pharmaceutical agents that can inhibit platelet function and prevent thrombotic events.

The intracellular juxtamembrane domains (JMD) of transmembrane, cell-surface proteins are rich in critical peptide sequences that participate in dynamic cell signalling events. Research from our group and others has shown that certain short linear synthetic peptides, based on highly conserved motifs in the JMD of cell-surface receptors, are able to selectively modulate the function of the parent receptor [1–4]. We therefore defined ‘Short linear motifs’ (SLiMs) as functional sites present in disordered regions of native proteins [5]. SLiMs can be identified using sequence alignment [6], or searches for occurrences of pre-defined motifs in protein sequences, to discover putative novel motif instances. Motif attributes known to be strong discriminators of motif functionality include accessibility and sequence conservation [7] and are based on structural, biophysical and biochemical features derived from the protein primary sequence [8]. Previously, Edwards *et al.* [9] identified SLiMs from disordered JMD regions of a panel of platelet proteins to evaluate if SLiMs might form a basis for developing new pharmacophores to target platelet function. In particular, one of the peptides derived from the JMD of the single-pass cell-adhesion protein, K-cadherin, was identified as an inhibitor of platelet function. K-cadherin was subsequently shown to be expressed in platelets and to play a role in thrombus formation [10].

Cadherins are Ca^2+^-dependent transmembrane proteins present on the cell surface that are involved in the formation of adherens junctions and intercellular recognition. The extracellular segments of cadherins form homophilic binding interactions with neighbouring cells, whereas the intracellular segments interact with catenins, such as P120-catenin, β-catenin and α-catenin [11]. Catenins connect the intracellular segments of cadherins with actin, thus controlling cytoskeletal changes and mechanophysical transformations of cells [12]. The interactions that involve cadherin–catenin complexes constitute the adhesion-dependent functions of cadherins. Cadherins also display adhesion-independent functions [13].

The role of cadherins in platelet function is not extensively characterized. Elrod *et al.* demonstrated the expression of E-cadherin in human platelets but its function was not reported [14]. Edwards *et al.* demonstrated a potential functional role for cadherin cell adhesion molecules in platelet function [9]. However, the function of cadherins in platelet-mediated events has not been widely studied.

In epithelial cells, E-cadherin represents a key molecule in the establishment and stabilization of cellular junctions [15]. Previously, we have shown that SLiM peptides derived from the JMD of E-cadherin act as TGFβ1 signalling modifiers in epithelial cells [16]. Therefore, we explored the bioactivity of cadherin-derived peptides in platelets to further elucidate the role of cadherins in platelet function and to determine the molecular mechanisms involved in these events. Our results identified that peptides derived from E-cadherin can potently inhibit platelet function in a sequence-dependent manner. This highlights a previously unknown role for the E-cadherin/P120-catenin axis in platelet function. Moreover, we have identified the minimal pharmacophore required for functional inhibitory action and have mapped the bioactive residues within inhibitory peptides.

## 2. Methods

### 2.1. Materials

Peptides were custom synthesized by Peptide v. 2.0, USA, with 90% purity (electronic supplemental material, table S1). Thrombin receptor activating peptide (TRAP) was purchased from BACHEM, Switzerland. P120-catenin antibody was purchased from Cell Signaling Technology UK. Platelet aggregation materials including siliconized test tubes and stir bars were purchased from BIO/DATA Corporation, UK. All general materials were purchased from Sigma-Aldrich Ireland except where stated otherwise.

### 2.2. Platelet preparation

Washed platelets (WPs) were prepared from blood samples collected from healthy donors who were free of medications known to affect platelet function for at least 10 days, as previously described [17]. Written, informed consent was obtained from all participants in line with the Declaration of Helsinki. In brief, venous blood was drawn into 15% (v/v) of acid-citrate-dextrose (ACD) anticoagulant (38 mM citric acid anhydrous, 75 mM sodium citrate, 124 mM dextrose). Blood was centrifuged at 150*g* for 10 min and the platelet-rich plasma (PRP) was collected. PRP was further centrifuged at 720*g* for 10 min in the presence of 1 µM prostaglandin E_1_ (PGE_1_). The platelet pellet was resuspended in buffer A (6 mM dextrose, 130 mM NaCl, 9 mM NaHCO_3_, 10 mM sodium citrate, 10 mM Tris base, 3 mM KCl, 0.81 mM KH_2_PO_4_ and 0.9 mM MgCl_2_·6H_2_O, pH 7.4). The washed platelet count was adjusted to 3 × 10^8^ platelets ml^−1^ and supplemented with 1.8 mM CaCl_2_ immediately prior to use.

### 2.3. Platelet aggregation

Platelet aggregation was monitored by light transmission aggregometry using a PAP-8 aggregometer (BIO/DATA Corporation, UK). WPs were stimulated with 4 µM TRAP at 37°C for 5 min under continuous stirring conditions. The effect of the peptides on platelet aggregation was assessed following pretreatment of platelets with 50 µM of peptide prior to aggregation for 12 min at 37°C. Data are expressed as the percentage of aggregation observed after stimulation of platelets with TRAP.

### 2.4. Platelet secretion

ATP secretion was measured using a luminescence-based assay, as previously described [18,19]. Peptides, buffer or vehicle control, in a final volume of 10 µl, were dispensed into white 96-well plates. To this, 70 µl of WPs were gently added. Platelets were incubated with peptides at 37°C for 12 min (under shaking) in the Perkin Elmer 1420 96-well plate reader. Platelet agonist (TRAP; 4 µM; 10 µl) was then added to induce platelet ATP secretion. The suspension (peptide + platelets + agonist) was incubated for 3 min at 37°C with constant shaking. Finally, 10 µl of ATP detecting reagent chronolume (Labmedics, UK) was dispensed into each well and luminescence was measured using the Perkin Elmer 1420 96-well plate reader. Platelet ATP secretion response to peptides was measured in the presence and absence of agonist. Data were expressed as the amount of ATP secretion in luminescence arbitrary units (arb. units). Data are represented as the mean ± standard error mean (s.e.m.) of at least four independent donors.

### 2.5. Platelet adhesion assay

Microtitre wells were coated with either bovine serum albumin (BSA) or fibrinogen (20 µg ml^−1^) at 4°C, overnight. Wells were blocked with 1% BSA in phosphate-buffered saline solution (PBS) for 2 h at 37°C and then washed three times with PBS. Aliquots (50 µl) of washed platelets without peptide or those preincubated with peptides for 12 min were then added to microtitre wells and incubated for 30 min at 37°C. The wells were then washed three times with PBS and adhered platelets were quantified using an acid phosphatases assay. A para-nitrophenyl phosphate (PNPP) substrate solution (100 µl of 70 mM sodium citrate, 30 mM citric acid, 0.1% Triton 100X and 5 mM of PNPP (Thermofisher, Ireland)) was added to each well for 2 h at 37°C. Incubation was stopped by addition of 0.1 M sodium hydroxide and the absorbance at 405 nm was read.

### 2.6. Protein extraction and western blot analysis

For protein analysis, 3 × 10^8^ platelets ml^−1^ of both resting and TRAP-activated platelets were lysed for 1 h at 4°C using a radioimmunoprecipitation assay (RIPA) buffer containing 50 mM Tris-HCl pH 7.4, 1% (v/v) Nonidet P-40, 150 mM NaCl, 1 mM Na_2_VO_4_, 1 mM NaF, 1 mM PMSF, and 1/100 dilution of protease inhibitor cocktail (Sigma). The protein concentration was determined using a Bradford [20] protein assay (BioRad, UK). Lysates were resolved using 10% SDS-PAGE (1 h at 100 V constant current) and immunoblotted with P120-catenin primary antibody followed by incubation with HRP-linked secondary antibody. Blots were developed by incubation with enhanced chemiluminescence ECL solution (Thermo Scientific, UK) and imaged using UVP's BioDoc-Imaging System.

### 2.7. Statistical analysis

Data were analysed using GraphPad Prism v. 6. Data represented as ± s.e.m. of individual donors. Significance was compared between each treatment of peptide+TRAP versus TRAP alone or TRAP+DMSO (vehicle control) using one-way ANOVA (Tukey's multiple comparisons test). For KEPLLP control peptides (figure 5*b*) significance was compared between scrambled peptide versus each individual peptide treatment.

## 3. Results

### 3.1. Identification of cadherin peptides

**Figure 1.**
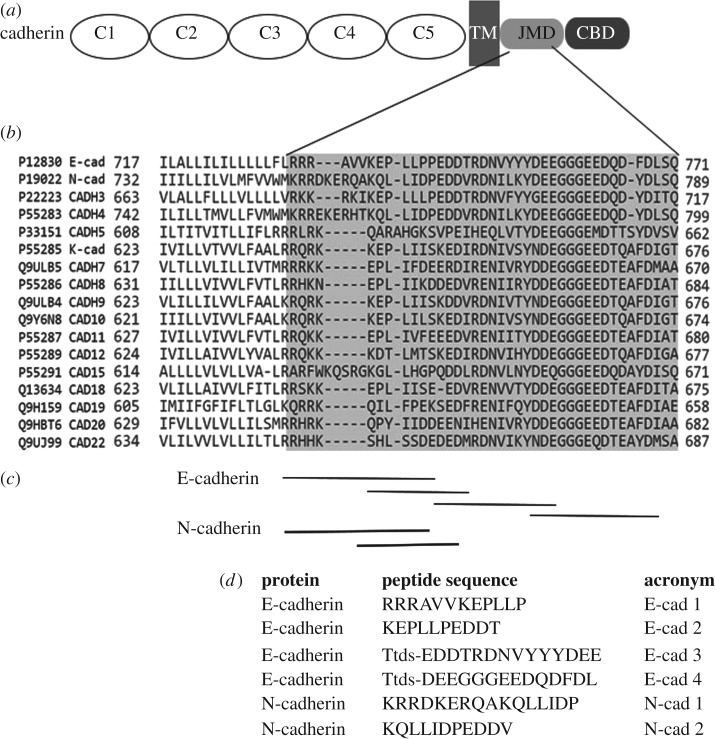
Initial set of test peptides derived from cadherins. (*a*) Graphical representation of the domain structure of human classical cadherins containing 5 cadherin-like extracellular domains (C1–C5), a transmembrane region (TM), juxtamembrane domain (JMD) which has P120-catenin interactions mapped to it, and the catenin binding region (CBD). (*b*) A zoomed-in image of the multiple sequence alignment of the JMD of different human cadherins. The P120-catenin binding region is highlighted in grey. (*c*) Peptide sequences designed from E- and N-cadherins are indicated below their position in the alignment and their sequences are shown in (*d*). All peptides are N-terminally palmitoylated (C_16_H_32_O_2_) and C-terminally amidated. E-cad 3 and 4 peptides were linked to Ttds (1-amino-4,7,10-trioxa-13-tridecanamine succinimic acid; C_14_H_28_N_2_O_6_) spacer between the N-terminal palmitate and the first amino acid.

In previous studies, we identified a short linear motif (SLiM) from cadherin that, when synthesized as a cell-permeable palmitoylated peptide, functionally interfered with platelet responses [9]. Here we designed overlapping peptides from E-cadherin and N-cadherin (figure 1) in order to localize the critical determinants of their inhibitory functionality. Figure 1 demonstrates the relative position of the peptides in the JMD of cadherin proteins. The same peptides are able to block TGFβ1-induced gene expression in epithelial cells [16].

Multiple sequence alignment of human cadherins (figure 1*b*) confirmed that cadherins are highly conserved in their JMD, especially within the P120-catenin binding region. Overlapping peptides from the JMD of E- and N-cadherins (figure 1*c*) were designed and synthesized. The peptides were N-terminally palmitoylated (pal) to facilitate tethering of peptide to the plasma membrane [1,3,9,21] and C-terminally amidated. A Ttds (1-amino-4,7,10-trioxa-13-tridecanamine succinimic acid) linker between the palmitic acid and the peptide sequence was used in E-cad 3 and E-cad 4 peptides to mimic in part the distance of the parent sequence from the plasma membrane. The peptides were named with letters and numbers. The letters refer to the parent cadherin (E and N) and the numbers refer to the position (rank) of the peptide relative to the plasma membrane. Adjacent peptides overlapped by 3–5 amino acids. Thus, the selected peptide from the region nearest to the membrane is referred to as peptide 1 (e.g. E-cad 1), with increasing peptide numbers corresponding to further distance from the membrane. Peptides sequences and acronyms are listed in figure 1*d*.

### 3.2. Cadherin peptides inhibit thrombin receptor activating peptide-induced platelet ATP secretion

TRAP is a 6-amino acid (SFLLRN) peptide that activates the platelet protease-activated receptor 1 (PAR1) and triggers platelet activation [22]. We evaluated the effect of cadherin-derived peptides on platelet dense granule secretion induced by TRAP. Platelet dense granules contain ATP, ADP and serotonin [23]. The quantitative effect of TRAP on platelet secretion was measured as the amount of ATP secreted using a reproducible high-throughput method [17,19]. TRAP induces a dose-dependent secretion of ATP that is maximal at 16 µM (electronic supplemental material, figure S1). A concentration of 4 µM TRAP was chosen for all experiments in this study as it induces a response that equates to approximately 80% of the maximal response. By using this sub-maximal dose, it is possible to identify both inhibitory and activating responses to peptides in our assays.

**Figure 2.**
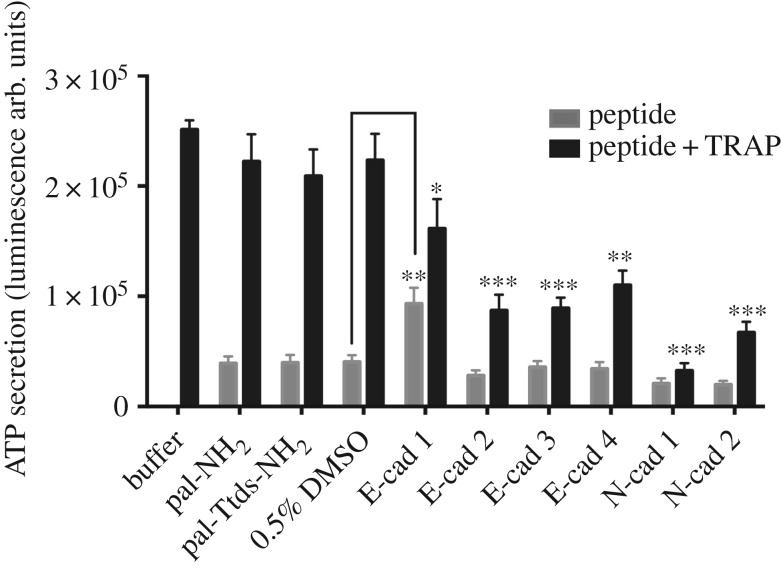
E- and N-cadherin-derived peptides inhibit platelet activation. Washed human platelets were preincubated with 50 µM of each peptide for 12 min at 37°C (grey bars). Following incubation, platelets were activated with 4 µM TRAP (black bars). Pal-NH_2_ was used as a control treatment in these experiments. Activation was measured as the amount of ATP released and expressed as luminescence arbitrary units (arb. units). *p < 0.05, **p < 0.01 and ***p < 0.001, One-way ANOVA compared with TRAP in the presence of palmitic amide (pal-NH_2_), for E-cad 1,2 and N-cad 1,2 peptides. For E-cad 3 and 4 peptides significance was compared to its control pal-Ttds-NH_2_. Significance between E-cad 1 peptide alone versus 0.5% DMSO alone was measured using Student's *t*-test, **p < 0.01. *N* = 6 individual experiments.

To investigate the effects of E- and N-cadherin peptides on the platelet function, washed human platelets were pretreated with various cadherin peptides prior to stimulation with TRAP. All cadherin peptides significantly inhibited TRAP-induced ATP secretion, particularly E-cad 2, N-cad 1 were the strongest inhibitors, and most peptides alone did not induce any platelet activation (figure 2). Longer incubation of E-cad 2 and N-cad 1 (for 1 h prior to TRAP stimulation) did not affect the peptide activity (electronic supplemental material, figure S2). Singularly, E-cad 1 peptide induced platelet ATP secretion, suggesting that this peptide could have both inhibitory and activating effects on platelets. In contrast, our controls (palmitic acid and palmitic acid connected to a Ttds linker) had no effect on basal or TRAP-induced platelet ATP secretion (figure 2). Having established the effects on ATP secretion of the E-cad 2 and N-cad 1 peptides, we then additionally tested their ability to alter the adhesion of platelets to immobilized fibrinogen. Platelets treated with E-cad 2 and N-cad 1 peptides failed to adhere to immobilized fibrinogen, suggesting that these peptides could affect platelet function other than ATP secretion (electronic supplemental material, figure S3).

### 3.3. Localization of critical determinants of peptide functionality

**Figure 3.**
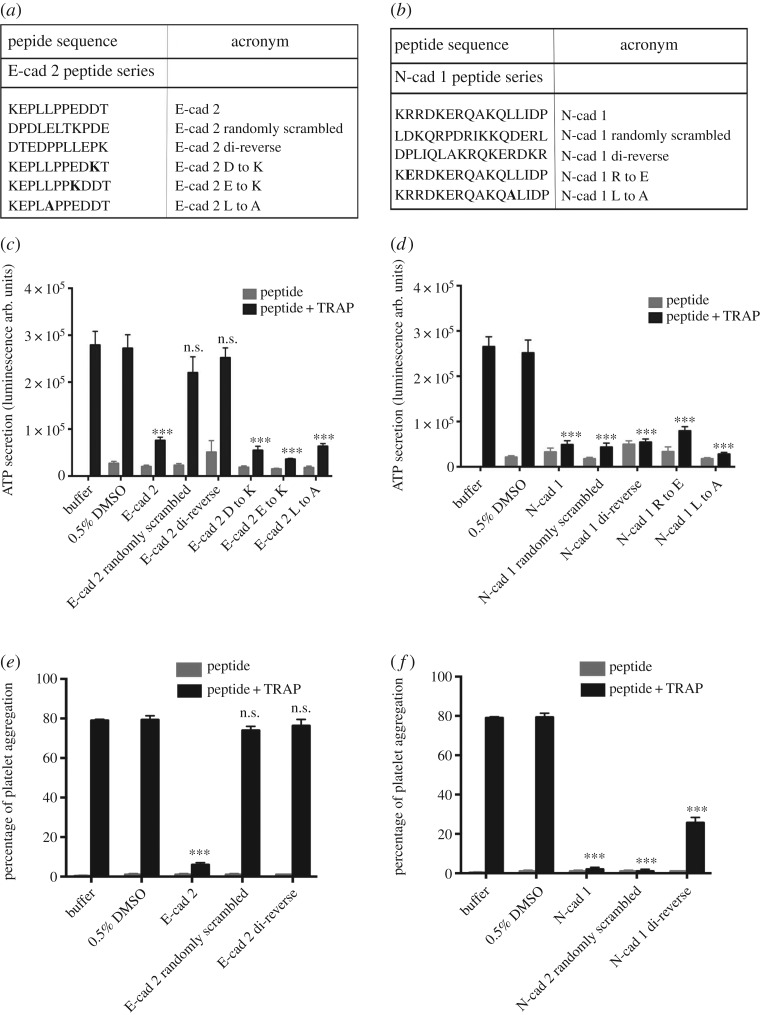
Effect of control peptides of E-cad 2 and N-cad 1 on platelet function. List of control peptides for (*a*) E-cad 2 and (*b*) N-cad 1. All peptides were N-terminally palmitoylated and C-terminally amidated. Amino acids indicated in bold in the list represent replaced residues within the peptide. A dose of 50 µM of peptides was used. Platelet secretion (*c*: E-cad 2; *d*: N-cad 1) and platelet aggregation (*e*: E-cad 2; *f*: N-cad 1) are shown. Significance was compared with platelets treated with TRAP in the presence of 0.5% DMSO versus each individual peptide treatment, calculated using one-way ANOVA. ***p < 0.001. Data represent mean ± s.e.m. of *n* = 6 individual donors for secretion and *n* = 3 for aggregation. n.s., non-significant.

Next, to further identify the role of cadherin peptides on platelet function we selected E-cad 2 peptide, which showed a potent inhibitory response among E-cadherin peptides. E-cad 2 peptide shares an overlapping sequence KEPLLP with E-cad 1 peptide (figure 1). Therefore, we hypothesized that there is a primary sequence present in one or both peptides that drives the inhibitory effect. Similarly, N-cad 1 peptide was chosen as it is closer to the membrane and appeared to be a potent inhibitor of TRAP-induced platelet ATP secretion, whereas a peptide further from the membrane, N-cad 2, was less effective. We employed various approaches to design control peptides to identify the sequence specificity of the chosen cadherin peptides. First, residues within the sequence were randomly scrambled (figure 3*a*,*b*). Next, we used a di-reverse approach where the order of the amino acids in pairs from C-terminus to N-terminus was reversed. For example, a control for KEPLLPPEDDT would be DTEDPPLLEPK (figure 3*a*,*b*). Finally, to identify the role of certain residues that were evolutionarily conserved compared to orthologues, specific amino acids were replaced with alanine as indicated in figure 3*a*,*b*.

The effects of control peptides were investigated using both ATP secretion and aggregation assays. We investigated the replacement of evolutionarily conserved amino acids. For example, the second aspartic acid (D) at the C-terminus of E-cad 2 peptide was replaced with lysine (K) ‘E-cad2 D to K’, and the first leucine (L) at the C-terminus of the N-cad 1 peptide was replaced with alanine (A) ‘N-cad 1 L to A’, and exhibited similar inhibitory activity on platelet function as the parent peptides (figure 3*c*,*d*). This observation suggests that evolutionarily conserved residues within the sequence (in orthologous proteins) are not individually crucial for biological activity in either peptide. E-cad 2 scrambled and di-reverse peptides failed to inhibit both platelet ATP secretion and aggregation, suggesting that activity of E-cad 2 is sequence specific (figure 3*c*,*e*). N-cad 1 scrambled and di-reverse peptides inhibited TRAP-induced platelet activation in both assays as potently as the parent peptide (figure 3*d*,*f*). Together these observations suggest that the E-cad 2 peptide exhibits sequence-specific activity. Aggregation responses mirrored those observed in the ATP secretion assay (figure 3*c*,*e*).

### 3.4. Mapping the bio-active residues within the E-cad 2 peptide

**Figure 4.**
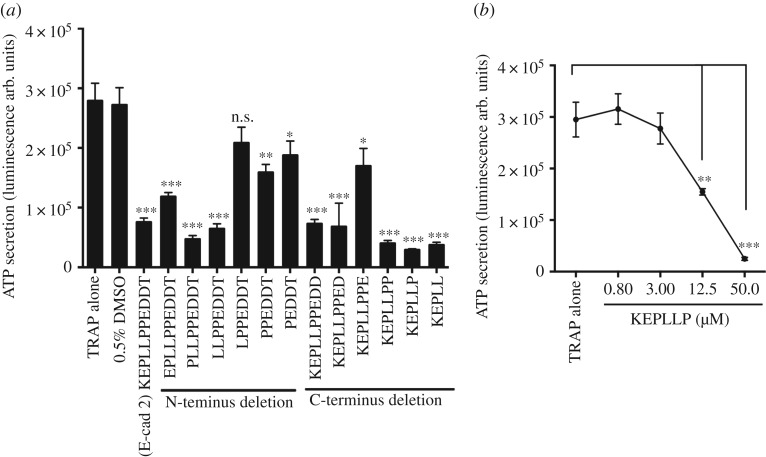
Identification of critical residues in E-cad 2 peptide. Effect of truncated peptides on platelet secretion (*a*) in the presence of TRAP 4 µM. Data represent ±s.e.m. of *n* = 6 individual donors. Dose-dependent effect of KEPLLP peptide (*n* = 4) (*b*). *p < 0.05, **p < 0.01 and ***p < 0.001 calculated using one-way ANOVA. Significance was compared between TRAP in the presence of 0.5% DMSO versus each individual peptide treatment. All peptides were N-terminally palmitoylated and C-terminally amidated.

The E-cad 2 peptide exhibited sequence-specific inhibition on platelet function. We aimed to identify the key residues responsible for this inhibitory effect. Truncated peptides were synthesized by systematic deletion of each residue from N- and C-terminus of E-cad 2 peptide. The effect of the truncated peptides on platelet function was assessed using the platelet ATP secretion assay. None of the truncated peptides activated platelets (data not shown). In contrast, most of the peptides continued to inhibit TRAP-induced platelet ATP secretion (figure 4*a*). Most of the inhibitory activity appears to be associated with the N-terminus of the peptide, as N-terminal deletions resulted in loss of activity. Notably, the short motif KEPLLP acted as the most potent inhibitor of platelet ATP secretion (figure 4*a*). To further confirm the specificity of KEPLLP peptide activity, we tested the effect of peptide with no palmitoylation. Non-palmitoylated KEPLLP peptide failed to inhibit the TRAP-induced platelet ATP secretion (electronic supplemental material, figure S4). This suggests that palmitoylation is necessary to direct the peptide towards the cell membrane. Next, to assess the potency of KEPLLP peptide activity, a dose–response study was performed. A 12.5 µM of KEPLLP was identified as the lowest peptide concentration that significantly inhibited TRAP-induced platelet ATP secretion (figure 4*b*).

### 3.5. Mapping the critical amino acids in KEPLLP peptide

**Figure 5.**
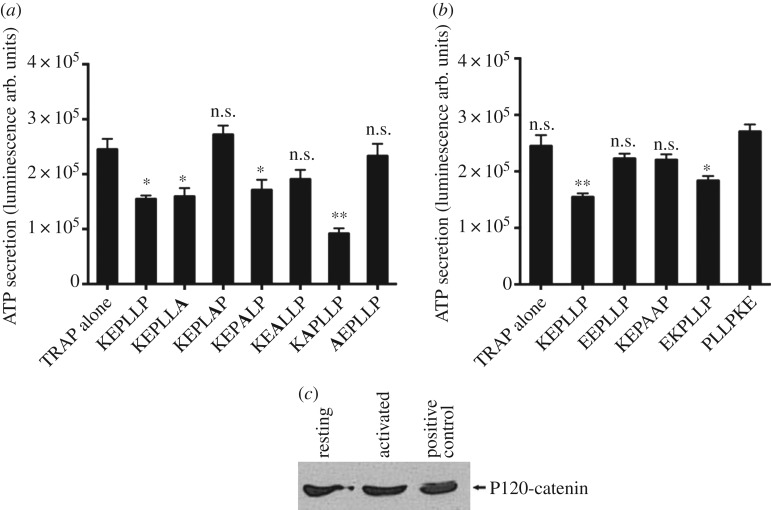
Identifying functional effects of the E-cadherin KEPLLP peptide in human platelets. (*a*) Replacement of each residue in KEPLLP peptide with alanine (highlighted in bold; significance was compared between each peptide treatment versus TRAP alone). (*b*) Control peptides of KEPLLP where lysine (K) replaced with glutamic acid (E), LL replaced with AA, reverse the charged amino acids at N-terminus (KE to EK) and scrambled peptide (PLLPKE). A 12.5 µM dose of peptides was used in the presence of TRAP 4 µM. *p < 0.05 and **p < 0.01 Student's *t*-test *n* = 4 individual donors. For KEPLLP control peptides, significance was performed using Mann–Whitney test between scrambled peptide versus each individual treatment. All peptides were N-terminally palmitoylated and C-terminally amidated. (*c*) Western blot analysis of P120-catenin in lysates of human resting and TRAP-activated platelets along with positive control (MDA-MB-231 cell lysate). Blot represents three different individual experiments.

KEPLLP sequence is an overlapping sequence in both the E-cad 1 and 2 peptides. Of note, catenins do not only bind to the CBD region (figure 1) but also bind to the JMD, with the KEPLLP motif lying in a ‘static’ binding domain for P120-catenin in E-cadherin [24]. In particular, the LL di-leucine motif appears to be crucial for the interaction of JMD of E-cadherin with P120-catenin [24]. Therefore, we hypothesized that the KEPLLP peptide is driving the observed inhibitory effect, and that the LL motif within the peptide is crucial for bioactivity. The hypothesis was tested by replacement of each residue with alanine (alanine scanning) to identify the key residues within KEPLLP. Replacement of one or both leucine (L) residues with alanine (A), to yield peptides KEPLAP and KEPAAP, resulted in loss of peptide activity (figure 5*a*,*b*). Alanine replacement of the proline (P) at the third position and the lysine (K) at the first position also resulted in loss of inhibitory activity (figure 5*a*). This suggests that these individual residues are crucial for bioactivity of this shorter peptide (although it is worth noting that the L5 replacement with A in a longer peptide did not lose inhibition, perhaps compensated by other residues within the peptide, see figure 3*c*).

We noted that replacement of the second residue E with A, which increased the positive charge of the peptide, also increased the inhibitory effect (figure 5*a*). We speculated that the N-terminal charge state might affect bioactivity, possibly by stabilizing interactions with negatively charged membrane. To identify the role of the positively charged lysine (K) at the first position of the KEPLLP peptide, we replaced it with the negatively charged glutamic acid (EEPLLP) and we observed loss of inhibitory effect (figure 5*b*). In addition, we synthesized the randomly scrambled peptide (PLLPKE) along with N-terminus reverse charge peptide (EKPLLP). The EKPLLP peptide retained partial inhibitory activity (figure 5*b*), this is not surprising since EKPLLP peptide still holds positive charged residue at N-terminus. However, fully scrambled peptide failed to inhibit platelet ATP secretion in response to TRAP (figure 5*b*). Thus, we can conclude that bioactivity of the KEPLLP peptide is sequence specific and that the positive charge of the N-terminus is important for peptide activity (figure 5*b*).

Expression of E-cadherin has been identified in platelets [14]. Cytoplasmic binding proteins of E-cadherin, β-catenin and α-catenin, are also found in platelets where they can form a functional complex with K-cadherin [25]. Moreover, β-catenin is involved in the Wnt signalling pathway in platelets [26]. Our peptides are derived from a P120-catenin binding region of E-cadherin. Of relevance, we identified expression of P120-catenin in both resting and TRAP-activated platelet lysates (figure 5*c*) indicating that the most likely interaction partner of the KEPLLP peptide is indeed present and available for interaction.

## 4. Discussion

Using a high-throughput platelet ATP secretion assay, we demonstrate that peptides derived from E- and N-cadherin inhibit TRAP-induced platelet dense granule secretion. We custom synthesized overlapping palmitoylated peptides from the JMD of E- and N-cadherins and tested their effect on platelet function. An E-cadherin peptide exhibited sequence-specific activity, which was primarily mapped to a short KEPLLP peptide. The inhibition of granule secretion by cadherin peptides suggests that these peptides may interfere with the function of upstream crucial signalling molecules involved in platelet granule release.

The design of control peptides is not straightforward, since random peptides may occasionally show activity or lose activity on the basis of biophysical properties that may not directly relate to peptide–target interactions of interest. Accordingly, we used several approaches to design various control peptides. This helped us to identify that the functional effects we observed from the chosen N-cad 1 peptide were not sequence specific, in contrast to those for the E-cad 2 peptide. These findings within our study of platelet function are consistent with findings of the effects of E-cad-2 peptide in epithelial cells, where it inhibited TGFβ1 signalling, Smad3 phosphorylation and Jagged expression in HK2 cells [16]. This raises the possibility that this KEPLLP cadherin region and its derived peptides may provide common mechanisms of action that may be amenable to therapeutic intervention, in both platelets and epithelial cells. However, the precise mechanism by which E-cad 2 peptide exerts its effect on platelet function remains to be determined.

To date, two studies have demonstrated that platelets express E-cadherin but its function remains unknown [14,27]. We observed expression of P120-catenin in platelets, a key regulator of E-cadherin function [25]. P120-catenin binds to the JMD region of E-cadherin and maintains cadherin stability at the cell surface [24,28]. Adhesion molecules not only participate in regulation of cell junctions but are also involved in intracellular signalling [13]. E-cadherin cross-talks with integrins in the progression of cancer, via several signalling molecules including Src, focal adhesion kinase (FAK) and Rho-family of GTPases [29]. P120-catenin is a key molecule for E-cadherin function and plays a role in the regulation of Rho-GTPases, which are involved in platelet function [30]. Interruption of E-cadherin/P120-catenin in platelets using the palmitoylated peptide derived from E-cadherin juxtamembrane domain could affect key signalling molecules involved in platelet secretion and aggregation. Based on this, it is tempting to speculate that E-cad 2 peptide may mimic the P120-catenin binding region, altering E-cadherin and P120-catenin interactions to consequently inhibit platelet function.

Experiments involving truncation of the E-cad 2 peptide demonstrated that residues at the C-terminus are most critical for the platelet-inhibitory activity. We identified the sub-peptide ‘KEPLLP’ as an inhibitor of platelet function. This sequence has been identified as a dynamic binding site for P120-catenin in E-cadherin JMD/P120-catenin interaction [24]. Interestingly, the LL motif within the KEPLLP peptide is involved in clathrin-mediated endocytosis of E-cadherin [31,32]. The binding of adaptor protein 2 (AP2) to the LL motif leads to clathrin-mediated endocytosis of E-cadherin [33]. AP2 is a heterotetramer consisting of two large adaptins (α or β), a medium adaptin (μ), and a small adaptin (σ) [33]. The association of P120-catenin with E-cadherin JMD may hide the LL motif from AP2, thereby preventing the internalization of E-cadherin from the cell surface. The KEPLLP peptide may well alter the distribution of E-cadherin and p120-catenin in the platelet on activation, and it will be of interest to determine if either of these proteins show substantial alterations in abundance or in their surface or internal localization, following peptide inhibition.

In summary, we have demonstrated that peptides derived from the JMD of E-cadherin can inhibit the activation of platelets, influencing both platelet aggregation and platelet secretion. The bioactivity of E-cad 2 peptide was narrowed down to a short sequence motif, KEPLLP, that is within the dynamic binding site of P120-catenin to E-cadherin.

## Supplementary Material

Supplementary Figures and Table

## Data accessibility

Underlying data have been publicly deposited and are available from the Dryad Digital Repository: http://dx.doi.org/10.5061/dryad.16m3n [34].
