# Supplementary material for: Peptides derived from cadherin juxtamembrane region inhibit platelet function
Source: R Soc Open Sci. 2018 Oct 10;5(10):172347. doi: 10.1098/rsos.172347 (PMC6227957; doi:10.1098/rsos.172347)
Supplement: Supplementary Figures and Table [file rsos172347supp1.pdf]

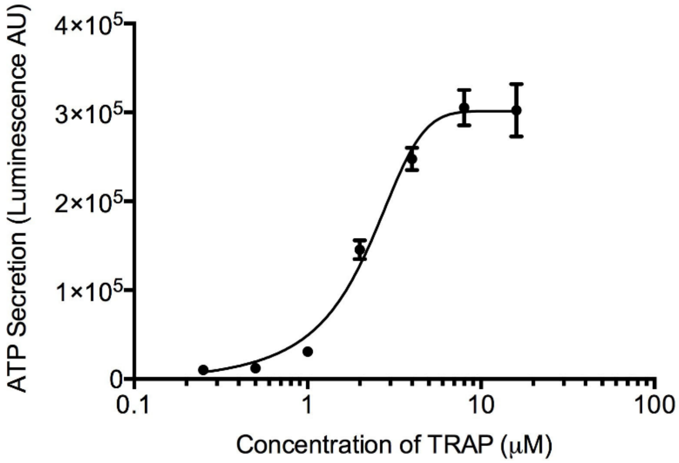

Supplemental figure I. Dose response of TRAP induced platelet ATP secretion. WPs were treated with various concentrations of TRAP (0.25μM to 16μM) for 3 minutes at 37°C. Amount of ATP secretion was measured using chronolume a luciferase reagent. Data expressed as luminescence arbitrary units. Data represents mean ±S.E.M of n=4 individual donors.

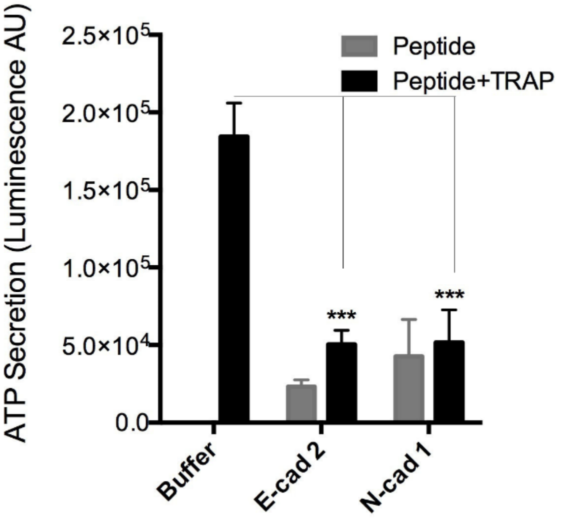

Supplemental figure II. Effect of longer incubation of E-cad 2 and N-cad 1 peptides on platelet secretion. WPs were incubated with 50μM peptide for 1hr at 37°C before stimulation with TRAP 4μM. Data represents ±S.E.M of n=4 individual experiments. ***P<0.001 calculated using Student's t-test.

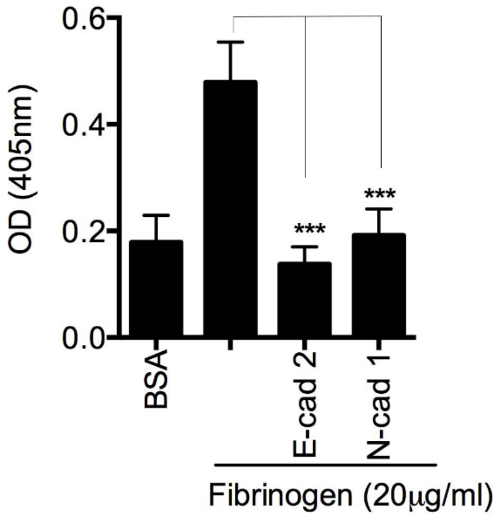

Supplemental figure III. E-cad 2 and N-cad 1 peptides significantly blocks platelet adhesion to immobilized fibrinogen. A 100μl of 3X10^{8} /ml of washed platelets (in the presence and absence of 50μM of E-cad 2 and N-cad 1 peptides) were added to wells coated with Fibrinogen. Amount of platelet adhesion was measured after 45 min incubation at 37°C using phosphatase assay following washing of unbound platelets. Data represents ±S.E.M of n=4 different individual experiments. ***P<0.001 student's t-test.

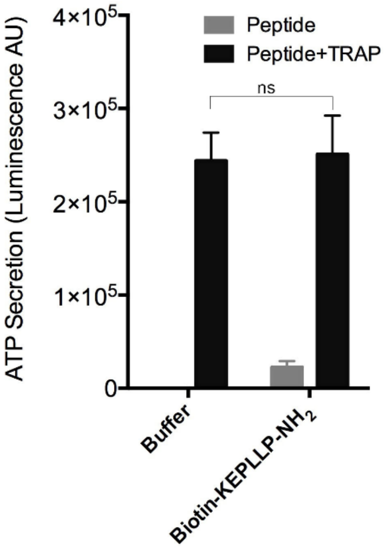

Supplemental figure IV. Non-palmitoylated KEPLLP peptide fail to inhibit TRAP induced platelet secretion. WPs were incubated with 50μM peptide for 12min at 37°C before stimulation with TRAP 4μM. Data represents ±S.E.M of n=4 individual experiments. Significance was calculated using Student's t-test.

A)

Acronym	Peptide sequence	Purity (%)
E-cad 1	RRRAVVKEPLLP	91
E-cad 2	KEPLLPPEDDT	91.3
E-cad 3	EDDTRDNYYYDEE	90.3
E-cad 4	DEEGGGEEDQDFDL	92
N-cad 1	KQLIIDPEDDV	90.5
N-cad 2	KRRDKERQAKQLLIDP	92.3
E-cad 2 Reverse charge	EKPLLPPKKKT	93
E-cad 2 Randomly Scramb	DPDLELTKPDE	97.3
E-cad 2 Di-reverse	DTEDPPLLEPK	91.3
E-cad 2 D to K	KEPLLPPEDKT	93
E-cad 2 E to K	KEPLLPPKDDT	91
E-cad 2 L to A	KEPLAPPEDDT	97
N-cad 1 Reverse chargege	EEEKEKEQAEQLLIKP	90.7
N-cad 1 Randomly scrambl	LDKQRPDRIKKQDERL	98.8
N-cad 1 Di-reverse	DPLIQLAKRQKERDKR	90.1
N-cad 1 R to E	KERDKERQAKQLLIDP	89.98
N-cad 1 L to A	KRRDKERQAKQALIDP	93.2
	EPLLPPEDDT	97.6
	PLLPPEDDT	89.9
	LLPPEDDT	95
	LPPEDDT	96
	PPEDDT	93.2
	PEDDT	96
	KEPLLPPEDD	91.7
	KEPLLPPED	91.09
	KEPLLPPE	91
	KEPLLPP	92.21
	KEPLLP	92
	KEPLL	91
	KEPLLA	95.3
	KEPLAP	95.71
	KEPALP	90.03
	KEALLP	95.45
	KAPLLP	91.38
	AEPLLP	90.34
	KEPAAP	90.05
	PLLPKE	91.07
	EKPLLP	90.07
	EEPLLP	89.95

B) Mass spectrometry (MS) data of KEPLLP peptide

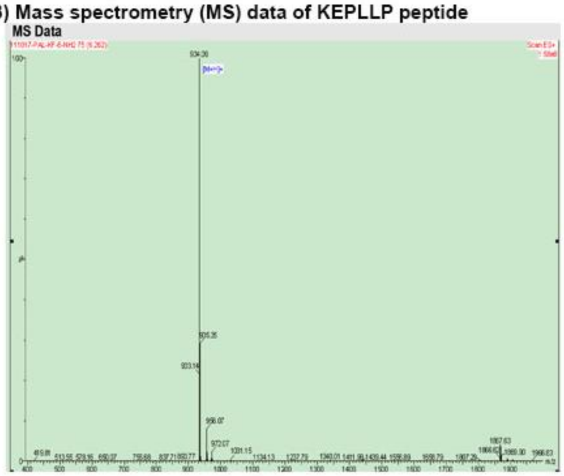

Supplemental table I: A) Information on peptides sequences and purity. B) MS data of KEPLLP peptide was shown as an example.
